# In Situ Time-of-Flight Mass Spectrometry of Ionic Fragments Induced by Focused Electron Beam Irradiation: Investigation of Electron Driven Surface Chemistry inside an SEM under High Vacuum

**DOI:** 10.3390/nano12152710

**Published:** 2022-08-06

**Authors:** Jakub Jurczyk, Lex Pillatsch, Luisa Berger, Agnieszka Priebe, Katarzyna Madajska, Czesław Kapusta, Iwona B. Szymańska, Johann Michler, Ivo Utke

**Affiliations:** 1 Laboratory for Mechanics of Materials and Nanostructures, Empa-Swiss Federal Laboratories for Materials Science and Technology, Feuerwerkerstrasse 39, CH-3602 Thun, Switzerland; 2 Faculty of Physics and Applied Computer Science, AGH University of Science and Technology Krakow, Al. Mickiewicza 30, 30-059 Kraków, Poland; 3 TOFWERK AG, Schorenstrasse 39, CH-3645 Thun, Switzerland; 4 Faculty of Chemistry, Nicolaus Copernicus University in Toruń, Gagarina 7, 87-100 Toruń, Poland

**Keywords:** ion extractor, mass spectrometry, electron induced fragmentation, lithography, TOFSIMS (Time-of-flight secondary ions mass spectrometry), FEBiMS, FEBID (Focused electron beam induced deposition), ice lithography, metalorganic compounds

## Abstract

Recent developments in nanoprinting using focused electron beams have created a need to develop analysis methods for the products of electron-induced fragmentation of different metalorganic compounds. The original approach used here is termed focused-electron-beam-induced mass spectrometry (FEBiMS). FEBiMS enables the investigation of the fragmentation of electron-sensitive materials during irradiation within the typical primary electron beam energy range of a scanning electron microscope (0.5 to 30 keV) and high vacuum range. The method combines a typical scanning electron microscope with an ion-extractor-coupled mass spectrometer setup collecting the charged fragments generated by the focused electron beam when impinging on the substrate material. The FEBiMS of fragments obtained during 10 keV electron irradiation of grains of silver and copper carboxylates and shows that the carboxylate ligand dissociates into many smaller volatile fragments. Furthermore, in situ FEBiMS was performed on carbonyls of ruthenium (solid) and during electron-beam-induced deposition, using tungsten carbonyl (inserted via a gas injection system). Loss of carbonyl ligands was identified as the main channel of dissociation for electron irradiation of these carbonyl compounds. The presented results clearly indicate that FEBiMS analysis can be expanded to organic, inorganic, and metal organic materials used in resist lithography, ice (cryo-)lithography, and focused-electron-beam-induced deposition and becomes, thus, a valuable versatile analysis tool to study both fundamental and process parameters in these nanotechnology fields.

## 1. Introduction

As a novel nanoanalytical method, focused-electron-beam-induced mass spectrometry (FEBiMS) relies on electron-stimulated desorption [1] of ionized fragments from matter and employs the focused electron beam of a scanning electron microscope (SEM) typically operated at high vacuum conditions >10^−6^ mbar. The ionized fragments, formed at the focus spot of the electron beam on the surface, are electrostatically channeled from the SEM chamber into a time-of-flight mass spectrometer (TOF-MS), enabling spatial in situ mass spectra acquisition. FEBiMS is an extension of the concepts of focused ion beam secondary ion mass spectrometry (FIBSIMS) [2,3,4]. The latter has recently developed into a powerful chemical characterization add-on tool for scanning focused ion beam microscopes, producing three-dimensional maps of elemental composition, with excellent spatial resolution, mass resolution, and sensitivity [3,5,6,7], also in combination with gas injection for signal enhancement [8,9,10,11]. FEBiMS covers a complementary field of interest, as it allows us to investigate the chemical interactions of electrons with matter in the range varying from 1 keV to few tens of keV energy (typically available in an SEM), not involving the removal of ionized material by mechanical sputtering cascades, as generated by ion beams. Electron irradiation activates various bond-breaking reactions, leading to the desorption of ions, neutrals, or radicals from surfaces [12]. These reactions play a fundamental role in astrophysics, radiation chemistry, or plasma physics and involve topics spanning from the cancer therapies to ozone or greenhouse gas to nanotechnology [13]. Furthermore, the possibility to measure the ionized volatile reaction fragments of electron interaction with matter enables fundamental chemical and material science and process studies related to the nanotechnology drivers in 2D and 3D structuring. These include thin film synthesis via electron-enhanced atomic layer deposition (EE-ALD) [14,15,16,17] and chemical vapor deposition (CVD) [18,19]; direct write 3D nanoprinting by gas assisted focused electron beam induced deposition (FEBID) [20,21,22,23]; and electron lithography with inorganic/metal organic resist materials [24,25,26,27], organic resists [28], ice lithography [29,30], and cryo-lithography with metal containing resists [31]. Furthermore, electron-induced reactions also play a vital role in extreme ultraviolet lithography [32] where the high-energy photons induce secondary electrons in metal-organic resists. The same holds for 3D nanoprinting by gas-assisted focused-ion-beam-induced deposition (FIBID) [33], wherein they determine the shape and composition of deposits, especially for noble gas FIBID performed in modern helium and neon ion microscopes [34,35]. This is due to the fact that primary light ions generate numerous secondary electrons as result of inelastic interactions with atoms. The electron-induced fragmentation reactions proceed via non-thermal excitation states of the (adsorbed) molecules. Charged fragments are created by dissociative ionization, dissociative electron attachment, and bipolar dissociation. There is presently no theory which can ab initio predict the fragment outcome of electron-induced fragmentation with molecules [36]. This specifically includes metalorganic compounds used in 3D nanoprinting by FEBID or FIBID (see reviews of Utke et al. [37]). and Barth et al. [38] Modeling of the fragmentation process is challenging and has so far been performed for diatomic and small polyatomic molecules only [39,40] on the base of experimental fragment information. The efficiency of dissociative electron attachment as a function of electron energy was simulated by resonance theory for a few molecules, e.g., HBr [41], isocyanic acid HNCO [42], methylformate HCOOCH_3_ [43], and acetylene C_2_H_2_ [44]. The efficiency of dissociative ionization has been modeled by the quasi-equilibrium theory for dimethyl silane (CH_3_)_2_SiH_2_ [45] by binary encounter Bethe formalism for small polyatomic atmospheric gases and hydrocarbons [46], or using the Deutsch-Märk (DM) formalism for noble gases [47]. Experimental approaches to explore the fragmentation behavior mechanisms of metalorganic molecules include ultra-high vacuum (UHV) gas phase [12,13,48,49] or condensed-phase investigations at cryogenic UHV conditions [50,51,52,53,54]. However, in their arrangements, they miss the specific conditions of electron irradiation, adsorption, and vacuum employed in focused-electron/ion-beam 3D nanoprinting or resist-based lithography processes.

In this study, we investigated the use of FEBiMS for direct writing in grains of metalorganic compounds and during FEBID. FEBiMS can be applied to in situ investigations of metalorganic resist lithography and the 3D FEBID nanoprinting process of metals. Metalorganic resist lithography proceeds via local irradiation of a condensed (non-volatile) compound on a substrate. The non-irradiated resist material is dissolved or evaporated after irradiating the desired pattern. Metalorganic precursors, such as MeCpPtMe_3_ [55,56] and W(CO)_6_ [57,58], as a condensed resist, were used in cryogenic conditions due to their high volatility. Non-volatile room-temperature metalorganic resist lithography so far comprises compounds of Pd [27,59,60], Ir [61], Au [26,62], Ag [63], and Cu [64]. Gas-assisted FEBID employs volatile precursors continuously supplied to the substrate via a gas injection system. The metal deposition occurs locally via electron-induced fragmentation of the precursor molecules adsorbed on the substrate surface to yield 3D structures. Non-irradiated adsorbed molecules desorb thermally from the surface and leave the SEM chamber via the exhaust system. The molecules of complexes are composed of organic ligands to render the metal volatile for delivery to the substrate inside the SEM vacuum chamber. A large variety of precursor families have been involved in FEBID studies, including metal carbonyls, β-diketonates, carboxylates, cyclopentadienyls, phosphines, halides, and alkyls [38,65]. However, FEBID and metal-organic resist approaches still suffer from relatively low metal contents in the deposited material [20]. One important bottleneck in further improving the metal content is the lack of information of the electron-induced mechanisms taking place during electron irradiation that would allow for us to pinpoint the inherent adsorbate fragmentation reactions versus secondary reactions determining the fate of the fragments. These include, for instance, ligands co-fragmentation or polymerization to non-volatile residues, which depend on the irradiation and vacuum conditions in the SEM chamber. Our FEBiMS measurements were performed under high vacuum conditions, similarly to the real FEBID and metalorganic resist lithography experiments. In situ FEBiMS monitors the outcome of electron-induced fragmentation within such conditions and can reveal details which are complementary to gas-phase and cryogenic condensed-phase studies performed under idealized conditions. Specifically, we present first in situ FEBiMS results for Cu_2_(µ-O_2_CC_2_F_5_)_4_, Ag_2_(µ-O_2_CC_2_F_5_)_2_, Ru_3_(CO)_12_ as metalorganic resists, and for FEBID with W(CO)_6_ gas injection.

## 2. Materials and Methods

### 2.1. FEB Induced Mass Spectrometry

**Figure 1:**
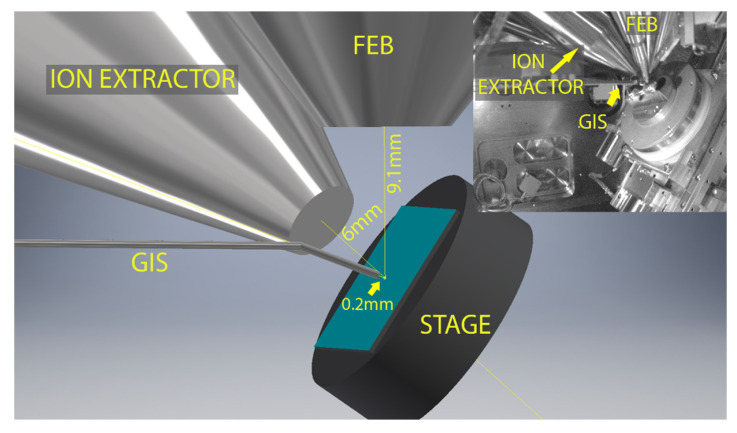
Schematic of the in situ FEBiMS experiment during FEBID with the gas injection system (GIS), the electrostatic lens ion extractor tube of the time-of-flight secondary ion mass spectrometer, the objective lens of the focused electron beam (FEB), and the sample stage with sample (s). The working distances are indicated. Inset: In-chamber view of measurement setup (infrared camera image). Note that there is no post-ionization involved in FEBiMS.

The focused-electron-beam-induced mass spectrometry experiments were performed with a TESCAN Lyra3 dual-beam system (Brno, the Czech Republic), consisting of a scanning electron microscope (SEM) and focused ion beam (FIB) equipped with a synchronized HV-compatible high-resolution TOF (HTOF) detector from TOFWERK (Thun, Switzerland) and commercial 5-line gas injection system (GIS) from Orsay Physics (Fuveau, France). The samples were irradiated with a 10 kV focused electron beam from the Schottky field emission gun, using beam currents of 5–30 nA. We want to highlight that, in contrast to standard quadrupole mass spectrometry employing post-ionization of gaseous fragments (e.g., in residual gas analyzers or in most experiments irradiating condensed precursor films or solid matter), FEBiMS relies on the direct extraction of the ionized fragments via the TOF extractor tube (which is an assembly of electrostatic lenses) at the point of their formation, namely close to the pixel where the primary electron beam impinges. The electric field was added by applying set of voltages on the electrostatic lenses of the TOF extractor. The voltage was optimized to maximize the obtained signal. Without an electrostatic-lens tube extractor to guide the ionic fragments to the TOF mass spectrometer, most of the ionic fragments are lost on the tube walls for detection, as they attach upon collision. Using a post-ionization scheme with a TOF-MS as the residual gas analyzer connected to the SEM was not successful. The electric field pulse duration in the TOF detector was set to 1000 ns to allow for detecting high mass species up to 450 *m*/*z*. The electron beam scan field (frame) was 20 µm × 20 µm and was divided into 256 × 256 pixels, which were then serpentine scanned with a pixel-to-pixel distance of about 78 nm and a dwell time of 40 µs per pixel. The mass spectrum was recorded at every pixel and can be binned according to the needs of the measurement. For our purposes, binning was performed for the entire frame, as we did not need the lateral mapping of composition. Figure 1 presents the in-chamber view of the setup (inset) and the geometry of the measurements. The substrate was 55° tilted with respect to the horizontal plane to maximize the number of ions that could reach the TOF extractor. The distances are given on the image. As the voltages of TOF ion extractor were optimized to work with GIS close to the sample, the GIS was located around 0.2 mm from the surface, even for the measurements that did not require using gases. Mass spectra data were collected and analyzed by using TOF-SIMS Explorer, version 1.12.2.0 software. The TOF-MS ion extractor can collect either positive or negative ions during the measurement. For measurements on grains of precursors, positive and negative ion spectra were thus recorded on the same grain and various grains to verify the reproducibility of measurements. The spectra were mass calibrated by using high-intensity peaks: *m*/*z* = 28 (CO^+^) and *m*/*z* = 186 (W^+^) for W(CO)_6_; *m*/*z* = 28 (CO^+^) and *m*/*z* = 32 (CH_3_OH^+^) for Ru_3_(CO)_12_; *m*/*z* = 31 (CF^+^) and *m*/*z* = 69 (CF_3_^+^/Ga^+^) for both carboxylates. The graphs were prepared by using Origin and graphics with CorelDraw, Adobe Photoshop, and AutoDesk Inventor.

### 2.2. FEBiMS on Solid Compounds

**Figure 2:**
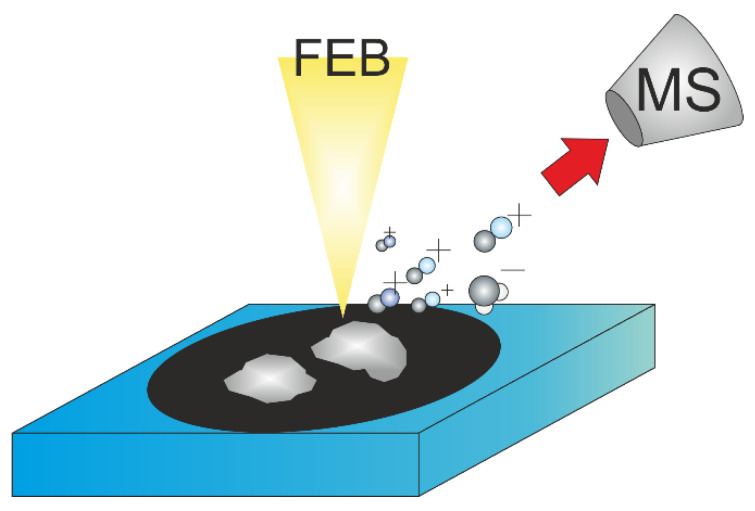
FEBiMS on solid compounds. The scheme shows non-volatile precursors’ grains fixed on carbon tape (black). The focused electron beam (FEB) fragments the compound molecules. Volatile ionized fragments are extracted to the mass spectrometer; neutral volatile fragments are pumped away. The 55° tilt of the sample and neutral fragments were omitted for clarity.

In this study, grains of complexes of ruthenium carbonyl Ru_3_(CO)_12_, silver pentafluoropropionate Ag_2_(µ-O_2_CC_2_F_5_)_2_, and copper pentafluoropropionate Cu_2_(µ-O_2_CC_2_F_5_)_4_ were placed on a conductive double-sided adhesive carbon tape from Ted Pella, and their excess was blown off with dry nitrogen gas to avoid chamber contamination. The Ru_3_(CO)_12_ precursor was purchased from Sigma-Aldrich. The silver and copper carboxylates were synthesized according to a process published by Szłyk et al. [66,67]. The schematics of the experiment are depicted in Figure 2. For each precursor, a different aluminum SEM stub and carbon tape were used to avoid cross-contamination and potential reactions between irradiated precursors. Several FEBiMS spectra were taken per compound to ensure reproducibility of the results.

### 2.3. FEBiMS Monitoring of FEBID

For FEBID with W(CO)_6_, boron p-doped (100) silicon wafer pieces with a native oxide layer were used as a substrate. To avoid organic surface contaminations (other than resulting from adsorption of residual gases from the SEM chamber), the Si pieces were cleaned subsequently with acetone for 5 s and twice in isopropanol (5 s each) in an ultrasonic bath. The samples were blown dry with N_2_. A 5-line GIS delivered the W(CO)_6_ through one line. The same scan parameters as used for FEBiMS (see Section 2.1) were applied. During the experiment, the W(CO)_6_ precursor reservoir was heated up to 90 °C. The gas lines were de-gassed at the same temperature prior to experiments. The substrate was kept at room temperature. For most of the samples (if it is not stated otherwise), prior to deposition, the GIS valve was closed, and the Si wafer was irradiated during 300 frames (300 correspond to about 15 min). The GIS valve was opened for the next 300 frames of irradiation (square deposition) and then closed during the final 300 frames for post-deposition irradiation. In total, a series of 4 or 5 squares, with ion detection using FEBiMS per sample, were deposited. EDXS and WDXS measurements were performed on 5 squares made during FEBiMS signal detection (for details, see next Section 2.4)).

### 2.4. Material Composition and Shape

The compositions of 5 FEB deposits performed with W(CO)_6_ were analyzed by using TESCAN Mira III (Brno, Czech Republic), scanning electron microscope equipped with EDAX systems for energy, and wavelength dispersive X-ray spectroscopy (EDXS and WDXS, respectively). The characteristic X-ray lines of Si-K (1.739 eV) and of W-M (1.775 eV) are overlapping in a standard EDX spectrum. Therefore, high-resolution WDS spectra were taken in the energy range between 1720 and 1820 eV. The analysis method combined standard analysis for W-M and Si-K lines with standardless analysis for C-K and O-K lines. Quantification results were corrected for thin film effects, using STRATAGem software. The WDS spectrum and results of quantification are available in Supplementary Figure S1.

The thickness of deposited structures was measured by using an NT-MDT (Moscow, Russia) atomic force microscope (AFM) in tapping (semi-contact) mode. Bruker RTESPA 300 AFM tips made of Si and covered with reflective layer of Al were used.

## 3. Results and Discussion

This section demonstrates the versatility of FEBiMS studies during electron irradiation and comprises observations on solid metalorganic compounds and monitoring of the FEBID process.

### 3.1. FEBiMS on Solid Compounds

The three solid low-volatility compounds, Ru_3_(CO)_12_, Ag_2_(µ-O_2_CC_2_F_5_)_2_, and Cu_2_(µ-O_2_CC_2_F_5_)_4_, were investigated, as schematically shown in Figure 2. All three metals present in the above complexes are important for nanotechnology and nanoscience. Ruthenium is used as a capping layer in novel EUVL masks, and as FEBID is used for mask repair [68], it is crucial to understand electron induced reactions in Ru complexes [54,69,70]. Pure silver nanostructures, due to their free electron gas, are desired in plasmonics and nanophotonics, e.g., surface-enhanced Raman scattering [71] or light-based information technology [72]. Copper, due to its low resistivity, has applications in micro- and nano-electronics. Direct-write nanostructuring of silver and copper is an ongoing topic, and carboxylates represent a novel precursor family for FEBID. Ag_2_(µ-O_2_CC_2_F_5_)_2_ and Cu_2_(µ-O_2_CC_2_F_5_)_4_ are stable compounds at ambient conditions at room temperature and were studied previously in FEBID [73,74,75,76], in metalorganic resist direct-write lithography [74], and in thermal CVD [77,78,79].

Although the experiments were performed in both positive- and negative-ions detection mode, for the precursors’ grains examined in this study, there were no statistically significant ion signals registered in the negative-ions mode (only a background noise), so only the positive-ions spectra are shown. For all of the samples, the influence of the unevenness of the surface of the grains on the measured signal was not considered. At this moment, it is hard to estimate what kind of influence this unevenness would carry; thus, more systematic study is needed to investigate it. The results were not spatially resolved; moreover, the measurement for each compound was repeated on several grains to check the reproducibility of the results, and no statistically important differences were noticed.

#### 3.1.1. FEBiMS on Ru_3_(CO)_12_ grains

Condensed-phase studies at cryogenic temperatures on other carbonyls W(CO)_6_ [52], Mo(CO)_6_ [80], Ni(CO)_4_ [81], and Fe(CO)_5_ [82,83,84] suggest the observation of the entire carbonyl ligand as CO^+^ (*m*/*z* = 28) in the mass spectrum. Similar studies on condensed Ru-carbonyl are missing. The mass limitation of *m*/*z* = 450 would include the detection of Ru_3_(CO)_5_ as the heaviest singly charged fragment and Ru_3_(CO)_12_ as the doubly ionized parent molecule. Ruthenium has five isotopes with relative abundance above 0.1, comprising *m*/*z* = 99, 100, 101, 102, and 104 (and two isotopes above 0.01 relative abundance with *m*/*z* = 96 and 98). For the mass spectrum, one can thus expect patterns of five neighboring peaks for any Ru-containing species.

**Figure 3:**
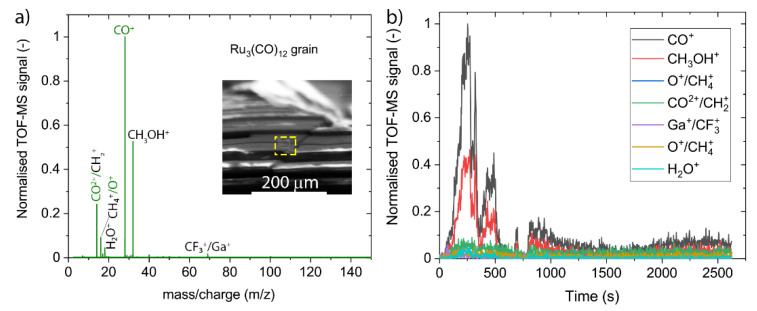
FEBiMS spectra of Ru_3_(CO)_12_ at 10 keV and 30 nA electron irradiation. (**a**) Accumulated spectrum over 2600 s. The inset shows a 55° tilt SEM picture of a part of the Ru_3_(CO)_12_ grain with the visible rectangular-shaped hole created during irradiation with electrons. (**b**) Time evolution of fragment peaks seen in (**a**).

The spectra presented in Figure 3 were acquired by using an electron beam at 10 keV energy and 30 nA current (spot size of around 700 nm) during around 44 min (1000 frames). The data were obtained in the mass-to-charge range varying between 1 and 450, but the heaviest measurable peaks appear at *m*/*z* = 69. Within the *m*/*z* ≤ 450 range, no Ru_3_(CO)^2+^_12−x_ nor Ru_3_(CO)^+^_1 to 5_ ESD fragments were detected, thus indicating that there is no electron-stimulated desorption of Ru-containing ions (the molecular mass of ruthenium is around 101 amu).

The most intense peak observed in the mass spectrum originates from CO^+^ ions (*m*/*z* = 28), whilst the third most intense peak corresponds to the detection of doubly ionized carbonyl ions CO^2+^ (*m*/*z* = 14). They suggest that carbonyl ligands detach and desorb during electron irradiation of adsorbed Ru_3_(CO)_12_ grains according to the reaction mechanism:

(1) $${\mathrm{Ru}}_{3}{\left(\mathrm{CO}\right)}_{12\;\left(\mathrm{ads}\right)}\overset{e-}{\to }{\;\mathrm{Ru}}_{3}{\left(\mathrm{CO}\right)}_{12-x}↓+\;\mathrm{xCO}↑$$

where upward- and downward-pointing arrows indicate desorbing and deposited species, respectively. This is consistent with the ESD results showing the detection of carbonyl ligands from few monolayer thin films of W(CO)_6_ and other carbonyls mentioned above, which were cryogenically condensed under UHV conditions. Furthermore, the spectrum in Figure 3 shows small peaks at *m*/*z* = 16 that may correspond to oxygen and can be attributed to the dissociation of the carbonyl ligand,

(2) $${\mathrm{CO}}_{\left(\mathrm{ads}\right)}\overset{e-}{\to }C↓+\;O↑,$$

leaving a non-volatile carbon as co-deposit to the ruthenium metal. Alternatively, CH_4_^+^ and CH_3_OH^2+^ exhibit the same *m*/*z* = 16 ratio and can be formed upon irradiation by the background residual water impinging and adsorbing on the solid Ru_3_(CO)_12_. The second most intense peak observed at *m*/*z* = 32 would point to CH_3_OH^+^ or O_2_^+^ fragment ions. The observation of the *m*/*z* = 18 peak (H_2_O) confirms the presence of residual water in this experiment. In contrast to Reaction 2, leading to the deposition of carbon, FEBiMS can hence propose the removal of carbonyl ligands or deposited carbon by methanol fragment formation should water be present in the system. The carbon removal is then likely performed by radicals formed by electron fragmentation of water adsorbates [85]. These radicals are highly reactive and form volatile compounds with the underlying carbon. Figure 3b demonstrates that the dynamics of the fragmentation process can be captured, including, for example, the rise of fragment intensity with irradiation time and the intensity decay after the irradiated volume has been fully fragmented into non-ESD material. The first time-evolution cycle suggests that fragmentation of the irradiated volume of Ru_3_(CO)_12_ was completed at around 400 s. The appearance of several rise/decay cycles, as seen in Figure 3b, was not investigated in detail. They may be attributed to shifts or drifts during the measurement due to charging. The presence of the lower intensity part, between 750 and 2500 s, may also be the result of such a drift or an interaction of backscattered electrons with the walls of the small crater created by scanning the area with the electrons.

#### 3.1.2. FEBiMS on Ag and Cu Carboxylate Grains

Ag_2_(µ-O_2_CC_2_F_5_)_2_ and Cu_2_(µ-O_2_CC_2_F_5_)_4_ were investigated in high-temperature FEBID and resulted in metal contents of around 25 at.% for Cu and 76 at.% for Ag which correspond to an elemental ligand loss of 80% to 95% upon electron irradiation with respect to the pristine molecule. Berger et al. [75] proposed, from EDX measurements, various fragmentation pathways for adsorbed Cu_2_(µ-O_2_CC_2_F_5_)_4_ ranging within:

(3) $${{\mathrm{Cu}}_{2}(µ-O_{2}{\mathrm{CC}}_{2}F_{5})}_{4\;(\mathrm{ads})}\;\overset{e-}{\to }2Cu↓+C_{2}F_{4}↓+3(O_{2}{\mathrm{CC}}_{2}F_{5})↑+CO_{2}↑+0.5F_{2}↑$$

(4) $${{\mathrm{Cu}}_{2}(µ-O_{2}{\mathrm{CC}}_{2}F_{5})}_{4\;(\mathrm{ads})}\;\overset{e-}{\to }2Cu↓+C_{2}F_{4}↓+C_{x}F_{y}↑+4CO_{2}↑$$

where upward- and downward-pointing arrows indicate desorbing and deposited species, respectively, and C_x_F_y_ being unsaturated and saturated fluorocarbons. Although these pathways were suggested from high-temperature FEBID experiments, they may also prevail at room temperature, as electron irradiation experiments of condensed Cu_2_(µ-O_2_CC_2_F_5_)_4_ yielded similar low copper contents [64]. The validation of specific fragments forming during interaction with the focused electron beam remains elusive, as EDX captured the composition of the deposited material only. It can be then expected that the mass spectrum of metal carboxylates during FEBID could include all the fragments shown in the above two reactions comprising the parent pentafluorocarboxylate (O_2_CC_2_F_5_) ligand or smaller sub-fragments of it, such as C_x_F_y_ fragments, and CO_x_ moieties. Peaks of hydrocarbon CH_x_ fragments may occur as a result of electron-induced reactions between the precursor and residual water gas from the vacuum chamber.

**Figure 4:**
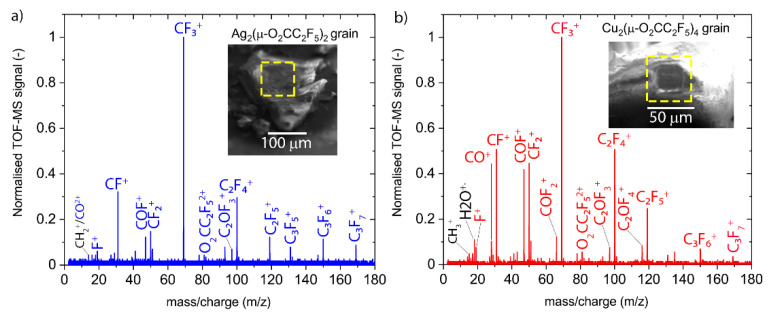
Accumulated FEBiMS spectra of grains obtained by 10 keV and 30 nA electron irradiation of (**a**) silver (I)-pentafluoropropionate and (**b**) copper (II)-pentafluoropropionate. Insets show the 55° tilt SEM pictures of irradiated grains with visible square-shaped holes created due to irradiation with electrons. Note that there is no mass peak at *m*/*z* = 163 corresponding to ESD of the singly charged parent carboxylate ligand fragment (µ-O_2_CC_2_F_5_), but there is a visible peak at *m*/*z* = 81.5 which corresponds to its doubly ionized state.

As presented in Figure 4, the mass spectra of both carboxylates are qualitatively similar. They show mostly the same peaks but with different relative intensities. However, the Cu_2_(µ-O_2_CC_2_F_5_)_4_ spectrum shows additional peaks in the low *m*/*z* region. The data were obtained in the mass-over-charge range between 1 and 450, with the heaviest visible peaks at *m*/*z*= 180.

The most prominent peak comes from CF_3_^+^ ions (*m*/*z* = 69). What can also be noticed is that both spectra exhibit the same series of peaks, coming from several fluorocarbons: CF^+^ (*m*/*z* = 31), CF_2_^+^ (*m*/*z* = 50), C_2_F_3_^+^ (*m*/*z* = 81), C_2_F_4_^+^ (*m*/*z* = 100), C_2_F_5_^+^ (*m*/*z* = 119), C_3_F_5_^+^ (*m*/*z* = 131), C_3_F_6_^+^ (*m*/*z* = 150), and C_3_F_7_^+^ (*m*/*z* = 169). These peaks can potentially confirm the fragmentation pathways proposed in Reaction 4, together with the fragment CO^+^ (*m*/*z* = 28) for the copper compound. The peak at *m*/*z* = 81.5 corresponds to the doubly charged parent carboxylate ligand fragment (O_2_CC_2_F_5_)^2+^ and can be expected from the fragmentation reaction 3. Furthermore, two peaks with oxyfluoro-carbon fragments are present: COF^+^ (*m*/*z* = 47, visible in both spectra) and COF_2_^+^ (*m*/*z* = 66, only Cu compound) in the silver compound spectrum. The copper compound spectrum contains more oxyfluoro-carbon fragments at *m*/*z* = 47, 66, 97, and 116, which may be attributed to the twice larger amount of ligands per copper atom compared to the silver compound. Of note is that the oxyfluoro-carbon fragments can be formed by the ligand elements themselves; however, they can also be a result of adsorbed water molecules from the background gas, which could be detected for the copper compound at *m*/*z* = 18. The source of the water is not clear presently. It might be contained in the compound or SEM background gas. What can be noted is the absence of CO_2_^+^ fragments in spectra of both carboxylates. This fragment was observed in recent electron-induced mass spectrometry (EI-MS) studies of irradiated layers of non-fluorinated silver carboxylates in UHV [86]. The lack of this fragment may indicate different electron-induced dissociation pathways for fluorinated and non-fluorinated silver carboxylates, thus possibly explaining the difference in metal content obtained in FEBID by using these group of precursors [74,87]. However, the lack of this fragment might also be caused by the difference in the experimental conditions between our study and EI-MS in UHV.

The time evolution of peak intensities is shown in Supplementary Figure S2. It shows a delay period of around 100 s for Cu_2_(µ-O_2_CC_2_F_5_)_4_ and almost 1000 s for Ag_2_(µ-O_2_CC_2_F_5_)_2_ after start of the irradiation before ionic fragments could be detected. The complete fragmentation of the irradiated volume was achieved after about 600 s for Ag_2_(µ-O_2_CC_2_F_5_)_2_ and 300 s for Cu_2_(µ-O_2_CC_2_F_5_)_4_.

### 3.2. FEBiMS Monitoring during W(CO)_6_ FEBID

**Figure 5:**
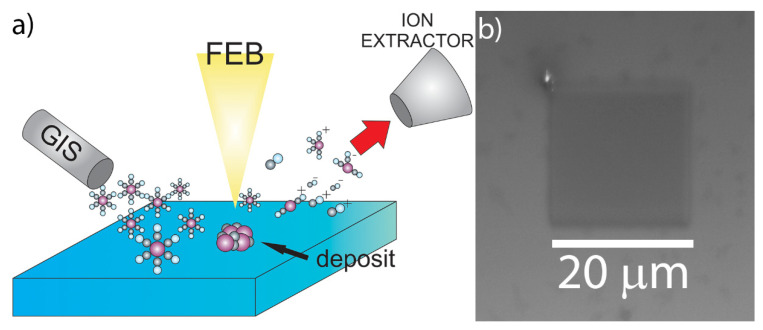
(**a**) Schematic of FEBiMS monitoring during FEBID. The precursor W(CO)_6_ is delivered through a GIS. The electron beam fragments the surface-adsorbed molecules and the non-volatile fragments form the FEBID deposit. Volatile ionized fragments are extracted to the mass spectrometer; neutral fragments cannot be detected and are pumped away. The 55° tilt of the sample and neutral fragments were omitted for clarity. (**b**) Top-view SEM image of the deposited square from W(CO)_6_.

The schematic setup of FEBiMS monitoring is shown in Figure 5a. Figure 5b shows the deposited 20 µm × 20 µm square. The deposit was composed of 1.5 at.% W, 87.5 at.% C, and 11 at.% O (see Supplementary Section S1). This tungsten content is low when compared to the literature values of FEBID, where purity was up to 30 at.% W [88], and for direct writing with Ga^+^ ions in the condensed layers of precursor, where purity was between 10 and 20 at.% [57]. However, we obtained the same composition of FEBID material in a series of experiments conducted without or with an inserted TOF ion extractor in negative- and positive-ion detection mode (see Supplementary Figure S3). In the scope of this article, we did not investigate further potential causes of the low metal content, such as precursor aging or irradiation conditions, which may have caused the low tungsten content.

Previous condensed-phase studies at cryogenic UHV conditions by Spencer et al. on metal carbonyl precursors [50] suggest that two surface reactions take place. The first reaction leads to the deposition of carbonyl-deficient metal carbonyl fragments, as it releases volatile carbonyl ligands from the parent molecules. The second reaction leads to the fragmentation of the residual carbonyl ligands. This fragmentation deposits carbon and releases oxygen that can either desorb, oxidize the metal, or react to volatile CO_2_ with the remaining carbonyl groups. Specifically for W(CO)_6_, Rosenberg et al. [52]. found that, on average, two carbonyl groups desorbed from condensed W(CO)_6_ by electron stimulation (500 eV), and that mainly WO_3_ and carbon were left as the final product of the further fragmentation of residual carbonyls upon further irradiation of the condensed phase:

(5) $$W{\left(\mathrm{CO}\right)}_{6\left(\mathrm{ads}\right)}\;\overset{e-}{\to }\;W{\left(\mathrm{CO}\right)}_{4}↓+2\left(\mathrm{CO}\right)↑$$

(6) $$W{\left(\mathrm{CO}\right)}_{4\left(\mathrm{ads}\right)}\;\overset{e-}{\to }{\mathrm{WO}}_{x}↓+4C↓+\left(4-x\right)O↑$$

where the upward- and downward-pointing arrows indicating desorbing and deposited species, respectively. FEBID experiments with W(CO)_6_ and 300 kV electrons performed by van Dorp et al. [89] indicate that electron irradiation lowers the activation energy for desorption by a factor of two to three compared to purely thermal desorption of W(CO)_6_. The authors concluded that the majority of the W(CO)_6_ molecules desorbed from the irradiated area rather than contributed to the deposition. Their method did not allow for the identification of the composition of ESD fragments.

#### 3.2.1. Mass Spectra

**Figure 6:**
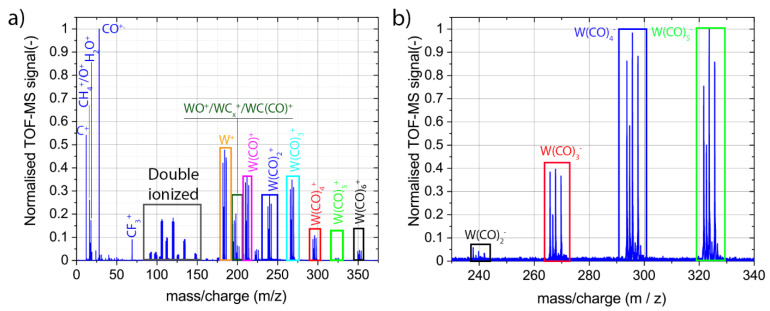
Mass spectra of ionized fragments registered during FEBID (10 kV, 30 nA) with W(CO)_6_ accumulated over around 40 min: (**a**) cationic fragments and (**b**) anionic fragments.

Figure 6 presents the positive and negative ion mass spectra collected by using FEBiMS during the FEBID experiment with the W(CO)_6_ GIS. The spectra were collected up to 450 *m*/*z*. As there were no mass peaks above 375 *m*/*z* for positive and above 340 *m*/*z* for negative spectra, only these parts are presented. Entire negative ion spectrum of W(CO)_6_ is presented in Supplementary Figure S4.

The positive ion spectrum in Figure 6a and the negative ion spectrum in Figure 6b indicate that the basic mechanism of fragmentation upon e-beam irradiation during FEBID with W(CO)_6_ is the cleavage of the metal–ligand bond, resulting in the loss of one or more carbonyl groups, as depicted in Reaction 5. As tungsten has four isotopes with significant abundance over 0.1 (*m*/*z* = 182, 183, 184, and 186), each W(CO)_x_ fragment shows four peaks in the mass spectrum. The peak intensities within one family correspond to the relative abundance of the tungsten isotopes for detailed mass spectra. It is interesting to note that all cation W(CO)_x_^+^ fragments, 0 ≤ x ≤ 6, are visible in the spectrum. The observation of the ionic parent ion W(CO)_6_^+^ is in line with the observation of electron-enhanced desorption of W(CO)_6_ of van Dorp et al. [89] discussed above. Moreover, anionic fragments of W(CO)_5_^−^, W(CO)_4_^−^, W(CO)_3_^−^, and W(CO)_2_^−^ can be distinguished. There are no signals from the negatively charged parent molecules, nor from W(CO)^−^.

There are also intermediate species present in the positive ion spectrum, such as WC(CO)^+^ (222 ≤ *m*/*z* ≤ 226), WC_2_^+^ (206 ≤ *m*/*z* ≤ 210), WC^+^, and WO^+^ ions (194 ≤ *m*/*z* ≤ 202, with the overlapping peak at *m*/*z* = 198). For all aforementioned detected singly charged cationic fragments, doubly ionized fragments were also observed. The doubly ionized WC(CO)_2_^2+^ fragment (125 ≤ *m*/*z* ≤127) can be seen, while the singly ionized fragmentWC(CO)_2_^+^ is not very visible. The mass spectrum of relatively light cations (i.e., *m*/*z* < 50) consists of mostly single peaks (Figure 6a), with the most intense peaks originating from ionized carbonyl ligands CO^+^ (*m*/*z* = 28) and ionized C^+^ fragments (*m*/*z* = 12). The single peak measured at *m*/*z* = 69 comes from the Ga^+^ or CF_3_^+^ ions. As there is no signal at *m*/*z* = 71 (another isotope of Ga), the signal comes most probably from CF_3_^+^. The low *m*/*z* part of the negative ion spectrum contains only three mass peaks corresponding to C^−^ (*m*/*z* = 12), O^−^ (*m*/*z* = 16), and F^−^ anions (*m*/*z* = 19) (see Supplementary Figure S5). The appearance of fluorine in the spectrum is attributed to its presence in the background gas, possibly due to chamber wall desorption from previous experiments, which employed XeF_2_ gas via the GIS lines.

The extraction of W^+^ and C^+^ fragments, as well as the W(CO)_6-x_ anion and cation fragments, during FEBID may come as a surprise, as tungsten and carbon are believed to be non-volatile and heavy W(CO)_6-x_ fragments that lose volatility with a decreasing amount of carbonyl ligands. However, in a cryogenic (40 K) condensed-phase study of Fe(CO)_5_ in ultra-high vacuum, Massey et al. [90] also extracted heavy Fe(CO)_5-x_ anions and cations (1 ≤ x ≤ 4) upon irradiation with electrons (energy 4–22 eV), including pure iron fragments. In addition, they observed carbonyl cations, as well as oxygen and carbon both as cations and anions. Of note is that, in Massey et al.’s study, no post-ionization was involved—which is similar to our FEBiMS setup. In contrast, the surface science study of Rosenberg et al. [52] involving the post-ionization of neutral fragments did not show any W-containing species desorbing from the substrate (see Reactions (5) and (6)). The exclusive detection of volatile CO fragments (and volatile species in general) in mass spectrometric experiments involving post-ionization may be reasoned by collisions and successive sticking (immobilization) of neutral and/or ionic fragments on vacuum chamber surfaces and tubing walls leading to the mass spectrometer. In non-post-ionization setups, ion extraction guides the fragments without wall collisions to the mass spectrometer. The mass spectra in Figure 6, together with Massey et al.’s work [90], would thus propose ESD of a large variety of less/non-volatile tungsten-containing fragments (including carbon fragments), in contrast to the simple Reactions (5) and (6). The final composition of the deposited material would then depend on the individual ESD efficiency of each fragment and may be tuned by the FEBID process parameters.

#### 3.2.2. Signal Origin—Gas Phase vs. Adsorbed Phase

An alternative interpretation of the origin of the heavy ionic fragments including tungsten can involve potential gas phase ionization. Metal ion signals during gas phase ionization of organometallic molecules by the electrons (primary, as well as secondary, electrons) were indeed observed [12,13,91,92]. Neustetter et al. [93] obtained spectra similar to the ones shown in Figure 6a for the irradiation of gaseous W(CO)_6_ with an electron energy of around 70 eV in a cross-beam experiment. They identified dissociative ionization as the most possible physical mechanism of fragmentation. The negative ion spectrum of Figure 6b could also be consistent with the spectra presented by Wnorowski et al. [94], who observed exactly the same ionized fragments and their relative abundance in a gas-phase cross-beam experiment with W(CO)_6_ and low-energy electrons. They attributed the appearance of negative ions to a dissociative electron attachment mechanism.

An estimation of the ionizing fragmentation events in the gas versus the adsorbed phase involves the simulation of the local gas pressure [95] to obtain the concentration of gas molecules and the estimation of the travel paths of the electrons in the gas and condensed phase. Supplementary Section S5 contains the details. For our experimental conditions, it turns out that ESD events from the adsorbed phase can be about 500 times more intense than dissociation events in the gas phase when assuming equal cross-sections for ionizing fragmentation in the adsorbed and gas phase $\sigma_{ads}/\sigma_{gas}=1$. To the best of our knowledge, there are no experimental data available on the absolute values of cross-sections of both the gas and adsorbed phase for W(CO)_6_ (see also the conclusion of de Vera et al. [96]). The paper of van Dorp [97] states that, compared to the gaseous state, the condensed phase possibly quenches some fragmentation channels, while it may activate others due to the vicinity of neighbor molecules. Overall, the quantification of the ratio $\sigma_{ads}/\sigma_{gas}$ cannot be provided currently to fully conclude on the origin of the ionic fragments. Should quenching and activation of fragmentation channels in the adsorbed phase balance for a given gas precursor molecule, then our aforementioned estimation would suggest a 500-times stronger signal from the adsorbed phase, based on concentration and path length arguments.

#### 3.2.3. Peak Evolution Monitoring during FEBID with FEBiMS

**Figure 7:**
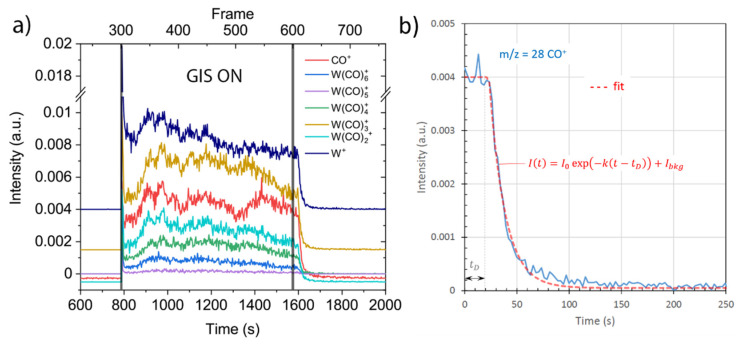
FEBiMS signal time evolution of selected peaks. (**a**) Time period including GIS opening, FEBID (GIS on), and GIS closing. The curves were separated on the vertical axis to increase readability of the data. (**b**) CO^+^ intensity decay after the GIS was closed (set to t = 0). The focused electron beam was continuously irradiating at 10 kV and 30nA.

Figure 7a represents the time evolution of signal intensities of selected peaks during the FEBID experiment, including the periods before and after GIS valve opening. The electron beam was switched on to stabilize and to be adjusted for focus and astigmatism before FEBID. The valve of the gas injection system was opened at frame 300, resulting in a rapid increase of intensities in all selected mass signals. During the next 300 frames, the GIS was open, and the 20 μm × 20 μm deposit was written on a substrate. After frame 600, the GIS valve was closed, and the signals all decreased to background level.

Figure 7b presents an exponential fit to the decay of the CO^+^ signal upon closing the GIS valve by using for the time-dependent MS intensity, $I\left(t\right)=I_{0}\exp\left(-k\left(t-t_{D}\right)\right)+I_{bkg}$, where *I_0_* is the average signal intensity before closing the GIS valve, *I_bkg_* is the background signal intensity, *k* is the decay rate (given by the ratio of pump speed and SEM chamber volume), and *t_D_* the delay time during which W(CO)_6_ gas still arrives through the GIS pipe system to the substrate. The fit parameters in Figure 7b were *I_0_* = 0.04, *I_bkg_* = 5·10^−5^, *k* = 0.067 s^−1^, and *t_D_* = 23 s. The value *k ^−^*^1^ = 15 s is a measure of the pump-down time it needs to reduce the gas concentration at the substrate surface to 1/*e* after GIS closure in our specific GIS/chamber/pump arrangement described in the experimental section, provided that there is linear dependence between the signal intensity and gas concentration. This value corresponds to the efficiency of the pumping system, the size of the GIS tube’s dead volume, the size of the chamber, and the temperature.

## 4. Conclusions

FEB-induced mass spectrometry is a powerful add-on method to a scanning electron microscope to screen the ESD fragments under typical SEM conditions of irradiation and high vacuum. Here we extracted ionized fragments (without post-ionization) during 10 keV electron irradiation of the metalorganic compounds of ruthenium carbonyl and silver and copper carboxylates: Ru_3_(CO)_12_, Ag_2_(µ-O_2_CC_2_F_5_)_2_, and Cu_2_(µ-O_2_CC_2_F_5_)_4_ For the ruthenium compound, the main dissociation mechanism was confirmed to be the loss of carbonyl ligands; however, the presence of C and O peaks in the spectrum indicates possibility of cleavage of the inter-carbonyl bond. For carboxylates, the presence of various fluorocarbons in the spectrum indicates that, despite the fact that the carboxylate ligand was heavily fragmented, the created moieties desorbed from the substrate, and this may explain the high ligand loss observed during FEBID with both compounds. The extraction of ionized fragments during gas-assisted FEBID revealed the existence of various tungsten–organic fragments including tungsten and thus a far more complex behavior than anticipated from cryogenic UHV condensed-phase studies. The origin of the ionized fragments tends to be electron-stimulated desorption from the adsorbed phase; however, a gas phase contribution cannot be excluded at this stage, as experimental values on absolute cross-sections are missing for a rigid conclusion. Further experiments would be needed to clarify the situation for FEBID monitoring.

FEBiMS can be extended to cryogenic stage cooling and ultra-high vacuum, as used in surface science. However, it can also straightforwardly perform at room temperature, high vacuum conditions, with specific gas ambience, and is thus easier and more straightforward to apply to fundamental issues concerning technological challenges. We therefore think that it is very interesting to apply to ice (cryo-)lithography with water and organic compounds, as well as for polymer-resist-based lithography, as it enables fundamental investigations at high electron beam fluxes, tens of kilovolt electron energies, and high vacuum conditions of 10^−6^ to 10^−5^ mbar typically used in these fields.

## Data Availability

The data is available on reasonable request from the first author.

## Supplementary Materials

The following supporting information can be downloaded at https://www.mdpi.com/article/10.3390/nano12152710/s1. Section S1: Composition of FEBID material. Section S2: Time evolution of mass spectrum for silver and copper carboxylates. Section S3: Study on influence of TOF electromagnetic field. Section S4: Entire negative ion spectrum of W(CO)_6_. Section S5: Estimation of gas phase vs. adsorbed phase signal. Figure S1. EDXS (black) and WDXS (red) spectra in the range between 1650 and 1950 eV of the typical deposit achieved during FEBiMS experiment; Figure S2. Signal time evolution of irradiated grains: (**a**) Ag_2_(µ-O_2_CC_2_F_5_)_2_ and (**b**) Cu_2_(µ-O_2_CC_2_F_5_)_4_. Time t = 0 indicates the start of irradiation with the FEB (10 keV, 30 nA); Figure S3. SEM top view image of FEBID square obtained from W(CO)_6_. The table shows the average composition of the sample in at.%, with 0.5 at.% of error margin prepared in each mode; Figure S4. Entire negative ion spectrum of W(CO)_6_ collected during FEBID experiment (10 keV, 30 nA); Figure S5. Simulated impinging gas flux in the FEBiMS arrangement. The gas injection nozzle is placed 200 μm above the substrate. The FEB impinges vertically (blue arrow) and secondary electrons are extracted to the SE detector (gray arrow).
