# Supplementary material for: In Situ Time-of-Flight Mass Spectrometry of Ionic Fragments Induced by Focused Electron Beam Irradiation: Investigation of Electron Driven Surface Chemistry inside an SEM under High Vacuum
Source: Nanomaterials (Basel). 2022 Aug 6;12(15):2710. doi: 10.3390/nano12152710 (PMC9370286; doi:10.3390/nano12152710)
Supplement: Supplementary file 1 [file nanomaterials-12-02710-s001.zip › nanomaterials-1796028-supplementary.pdf]

nanomaterials

Supplementary Materials

In Situ Time-of-Flight Mass Spectrometry of Ionic Fragments Induced by Focused Electron Beam Irradiation: Investigation of Electron Driven Surface Chemistry inside an SEM under High Vacuum

Jakub Jurczyk [1,2,†], Lex Pillatsch [3], Luisa Berger [1], Agnieszka Priebe [1], Katarzyna Madajska [4], Czesław Kapusta [2], Iwona B. Szymańska [4], Johann Michler [1] and Ivo Utke [1,*]

Laboratory for Mechanics of Materials and Nanostructures, Empa-Swiss Federal Laboratories for Materials Science and Technology, Feuerwerkerstrasse 39, CH-3602 Thun, Switzerland; jakub.jurczyk@unizar.es (J.J.); luisa.berger@epfl.ch (L.B.); agnieszka.priebe@empa.ch (A.P.); johann.michler@empa.ch (J.M.)

[2] Faculty of Physics and Applied Computer Science, AGH University of Science and Technology Krakow, Al. Mickiewicza 30, 30-059 Kraków, Poland; kapusta@agh.edu.pl

[3] TOFWERK AG, Schorenstrasse 39, CH-3645 Thun, Switzerland; lex.pillatsch@tofwerk.com

[4] Faculty of Chemistry, Nicolaus Copernicus University in Toruń, Gagarina 7, 87-100 Toruń, Poland; 502533@doktorant.umk.pl (K.M.); pola@umk.pl (I.B.Sz.)

* Correspondence: ivo.utke@empa.ch

† Current affiliation: Instituto de Nanociencia y Materiales de Aragòn, CSIC-Universidad de Zaragoza, C/Pedro Cerbuna, 12, 50019 Zaragoza, Spain

S1. Composition of FEBID Material

Figure S1 presents the average composition of square deposits made during FEBiMS experiments. The composition was measured by using a combination of energy dispersive X-ray spectroscopy (EDXS) and wavelength dispersive X-ray spectroscopy (WDXS) methods, because the characteristic X-ray lines WM (1775 eV) and SiK (1739 eV) overlap in a standard EDX spectrum. Standardless EDXS gave the oxygen and carbon content (for O and C). WDXS gave the W content, using pure metallic standard from Ted Pella(Redding, USA) for calibration. EDXS and WDXS were performed with 4 kV acceleration voltage. Five square deposits made in comparable conditions (electron energy of 10 keV and beam current of 8.9 nA). The scan strategy for FEBID was described in the experimental section of the manuscript (see Section 2.1) and comprised irradiation of the sample with serpentine strategy and the dwell time of 40 μs per pixel over an area of 20 μm × 20 μm, divided into 256 × 256 pixels (which resulted in point pitch of 78 nm). Prior to deposition, the GIS valve was closed, and the Si wafer irradiated during 300 frames (300 correspond to about around 15 min). The GIS valve was opened for the next 300 frames of irradiation (square deposition) and then closed during the final 300 frames for post-deposition irradiation (see Section 2.3). There were also three squares deposited without pre- and post-deposition irradiation to check their influence on metal content. The composition of these deposits differed only slightly, achieving 2.5 at.% of W, 88.5 at.% of C, and 9.0 at.% of O. All measurements have an uncertainty on the level of 0.5 at.%. The uncertainty was estimated as a maximal standard deviation of measurements taken from different squares made with the same deposition parameters.

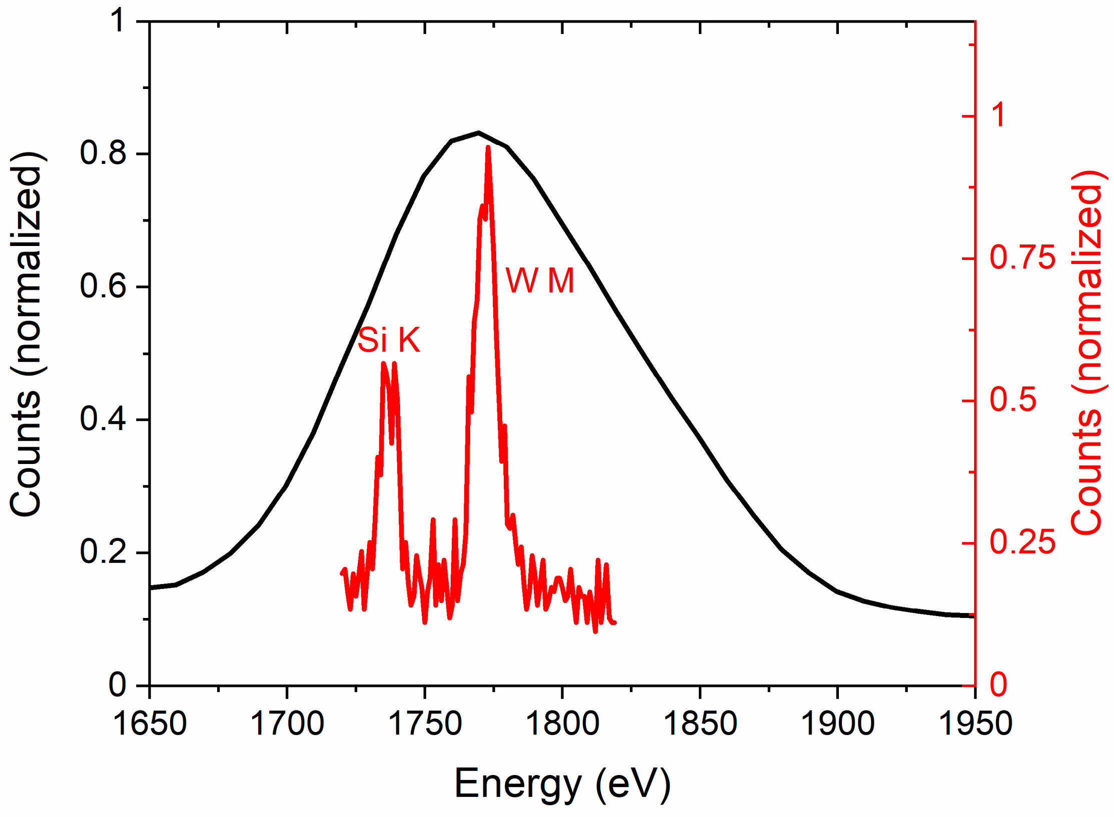

Figure S1. EDXS (black) and WDXS (red) spectra in the range between 1650 and 1950 eV of the typical deposit achieved during FEBiMS experiment.

Table S1. Elemental composition of the typical FEBiMS deposit.

Element	Atomic %
**W**	1.5
**O**	11.0
**C**	87.5

S2. Time Evolution of Mass Spectrum for Silver and Copper Carboxylates

The time evolution of peak intensities is shown in Supplementary Figure S1. They show a delay period of around 100 s for $Cu_2(\mu\text{-}O_2CC_2F_5)_4$ and almost 1000 s for $Ag_2(\mu\text{-}O_2CC_2F_5)_2$ after the start of the irradiation, before ionic fragments could be detected. The complete fragmentation of the irradiated volume was achieved after about 600 s for $Ag_2(\mu\text{-}O_2CC_2F_5)_2$ and 300 s for $Cu_2(\mu\text{-}O_2CC_2F_5)_4$. The fragment intensity oscillates for both compounds, indicating that the fragmentation may not be continuous with time or that drifts occurred due to charging.

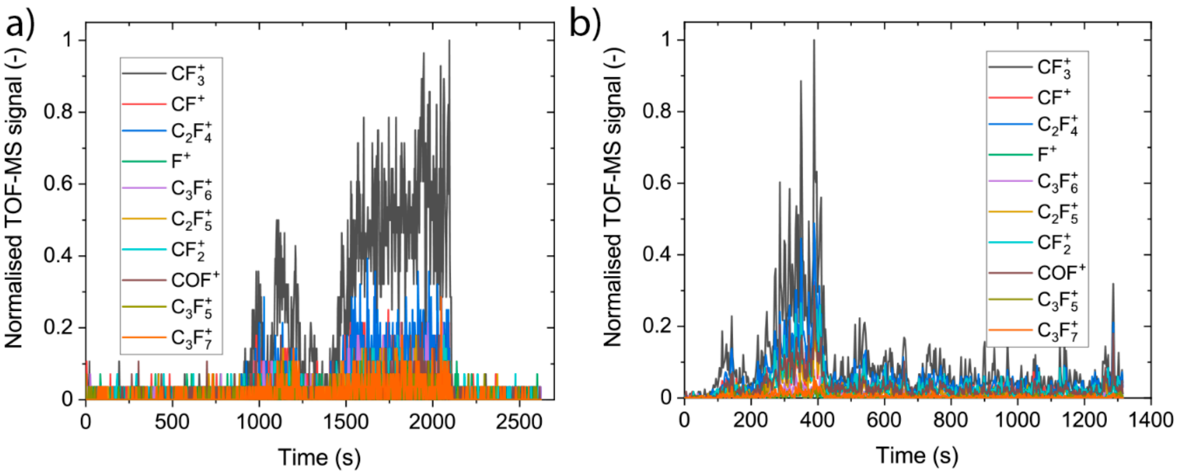

Figure S2. Signal time evolution of irradiated grains: (**a**) $Ag_2(\mu\text{-}O_2CC_2F_5)_2$ and (**b**) $Cu_2(\mu\text{-}O_2CC_2F_5)_4$. Time t = 0 indicates the start of irradiation with the FEB (10keV, 30nA).

S3. Study on Influence of TOF Electromagnetic Field

To rule out a potential influence of the electric fields created by the ion extractor and the time-of-flight mass spectrometer on the composition and growth rate of the deposits, a series of deposition experiments were performed by using the TOF system (ion extractor and TOF-MS) in three operation modes: (i) positive ion detection, (ii) negative ion detection, and (iii) no operation. These three operation modes include different settings of voltages applied to the ion extractor and the TOF-MS which may influence the electron beam. The no operation mode does not apply extraction voltages to the ion extractor.

All of the square deposits were written by using the same focused electron beam voltage of 10 kV, beam current of 5.2 nA (measured inside Faraday cup), working distance of 9.1 mm, and distance between the sample and the GIS. The scan strategy for FEBID was described in the experimental section of the manuscript. Prior to deposition, the GIS valve was closed, and the Si wafer irradiated during 300 frames. The GIS valve was opened for the next 300 frames of irradiation (square deposition) and then closed during the final 300 frames for post-deposition irradiation. The ion extractor was always inserted to avoid any differences in the geometrical factor. In total, seven 20 µm × 20 µm squares were deposited in the following order (see also Figure S3):

1. Three squares without any voltage applied to the TOF system;

2. Two squares with voltages for positive mode detection of the TOF system;
3. Two squares with voltages for negative mode detection of the TOF system.

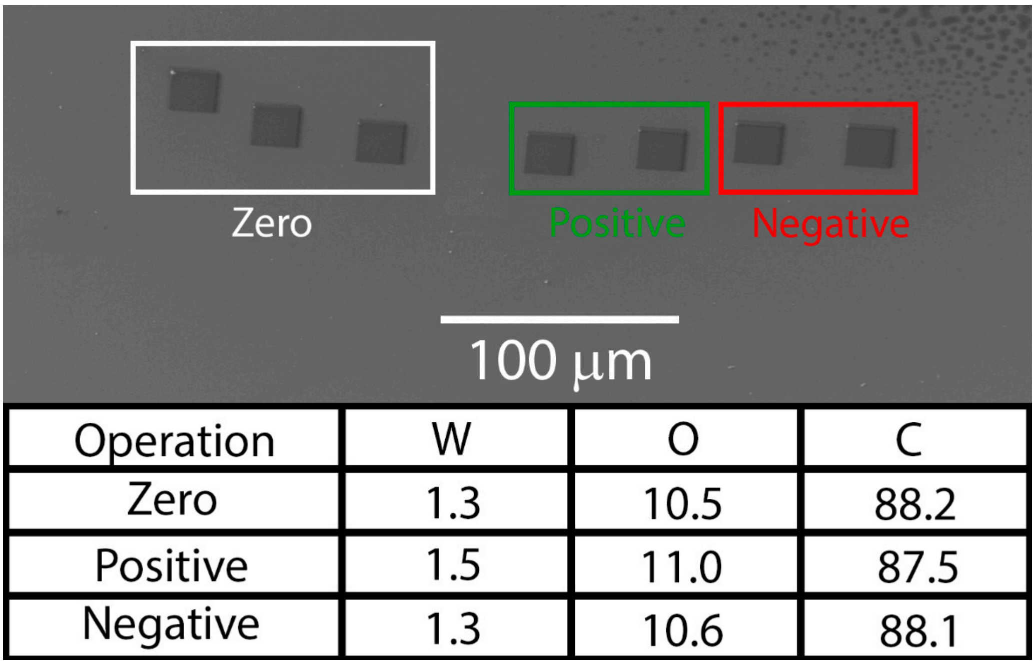

Operation	W	O	C
Zero	1.3	10.5	88.2
Positive	1.5	11.0	87.5
Negative	1.3	10.6	88.1

Figure S3. SEM top view image of FEBID square obtained from $W(CO)_6$. The table shows the average composition of the sample in at.%, with 0.5 at.% of error margin prepared in each mode.

Figure S3 presents the results of the combined EDXS and WDXS quantification (described in the experimental section of the article). There is no significant difference in composition for the three TOF system operation modes. This suggests that the electrostatic fields generated to extract the ionic fragments to the TOF-MS do not influence the composition of the FEBID material.

S4. Entire negative ion spectrum of $W(CO)_6$

Figure S5 presents the accumulated full-range negative ion spectrum recorded during $W(CO)_6$ FEBID, including the pre-deposition and post-deposition irradiation step (each step 300 frames with GIS closed). The four most intense groups of peaks, representing W-containing ionic fragments, were described in detail in the main article (see Figure 6b). There are two single peaks at the low m/z range which are visible in the extended spectrum of Figure S5. The peak at m/z=16 corresponds to O^- (or CH_4^-) ionic fragments, and the m/z=19 peak matches F^- fragments. Fluorine most probably originated from the residual gases of the SEM chamber, as XeF_2 is frequently used in this SEM for sample etching or FIBSIMS experiments. The spectrum was normalized to the highest intensity peak.

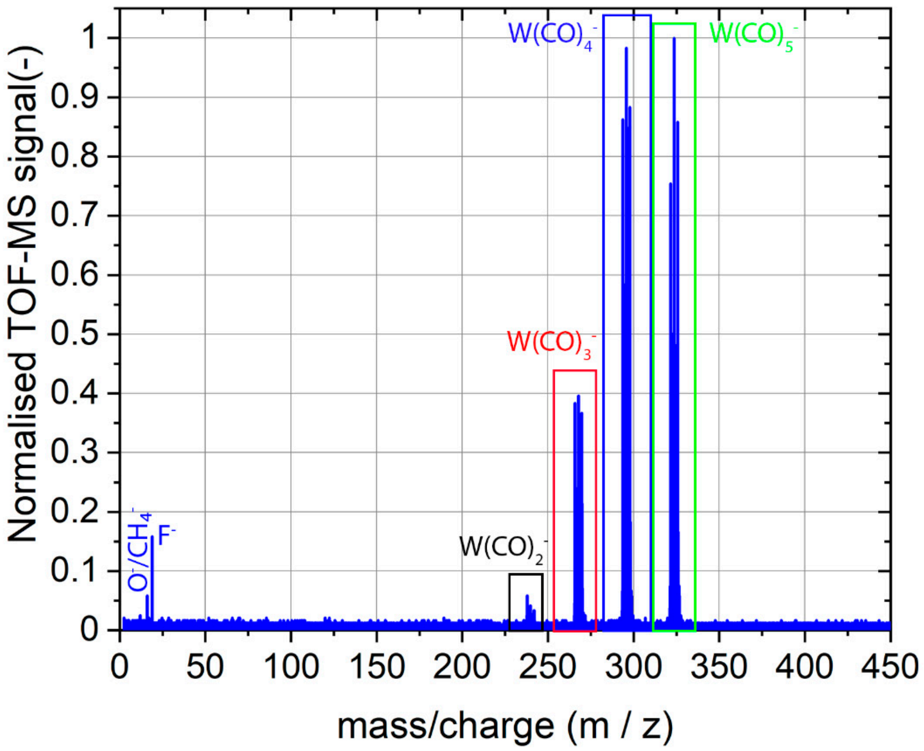

Figure S4. Entire negative ion spectrum of W(CO)$_6$ collected during FEBID experiment (10 keV, 30 nA).

S5. Estimation of gas phase vs. adsorbed phase signal

Ionization events, I, are proportional to the molecule concentration per volume, n, the ionization cross-section, σ, and the length of the path, l, the electron beam traverses the volume filled with molecules, $I \sim n\sigma l$. The molecule concentration for the gas phase is $n_{gas} = p/kT$, with p as the pressure, k as the Boltzmann constant, and T as the temperature. For the condensed phase, the concentration is $n_{ads} = \rho N_A/M$, with ρ as the density, N_A as the Avogadro constant, and M as the molar mass. The background pressure inside the FIBSEM chamber was around 2×10^{-6} mbar. The chamber pressure was shortly about 1×10^{-5} mbar right after opening of GIS valve and stabilized at around to 6×10^{-6} mbar during FEBID. Using the relation $= S \cdot \Delta p$, gives the precursor throughput, Q, through the GIS, with S as the net pumping speed and Δp as the pressure difference to the background pressure. Dividing the throughput by the nozzle exit area yields the molecule flux, J, which can be converted into the local pressure, P, at the nozzle exit via the well-known relation $J = PN_A(2\pi MRT)^{1/2}$, with N_A as the Avogadro constant, R as the universal gas constant, and T as the temperature. Entering the natural constants and experimental values yields a local pressure at the GIS nozzle exit of 9.5 Pa, which corresponds to $p = 5 \times 10^{-3}$ mbar on the substrate position where the FEB impinges (see Figure S6, below). We then enter other parameters, namely $T = 100$ °C (reservoir and nozzle temperature), $\rho = 2.65$ gcm^{-3}, and $M = 351.85$ g/mol, and the concentration ratio becomes $n_{ads}/n_{gas} \approx 5 \times 10^7$. As the ESD of ionized fragments from the adsorbed phase will probably not occur from a depth deeper than the two first monolayers, the travel path of the electron in the adsorbed phase can be taken as $l_{ads} \approx 1$ nm, which is about the size of a metalorganic molecule. The travel path of the electron in the gas phase can be estimated from Figure S5 as $l_{gas} \approx 100\ \mu m$. This implies an electron path

ratio of $l_{ads}/\ l_{gas} \approx 1 \times 10^{-5}$. For the adsorbed versus gas phase ratio of ionization events, this amounts, in our experimental conditions, to $I_{ads}/I_{gas} \approx 5 \times 10^{2} \cdot \sigma_{ads}/\sigma_{gas}$.

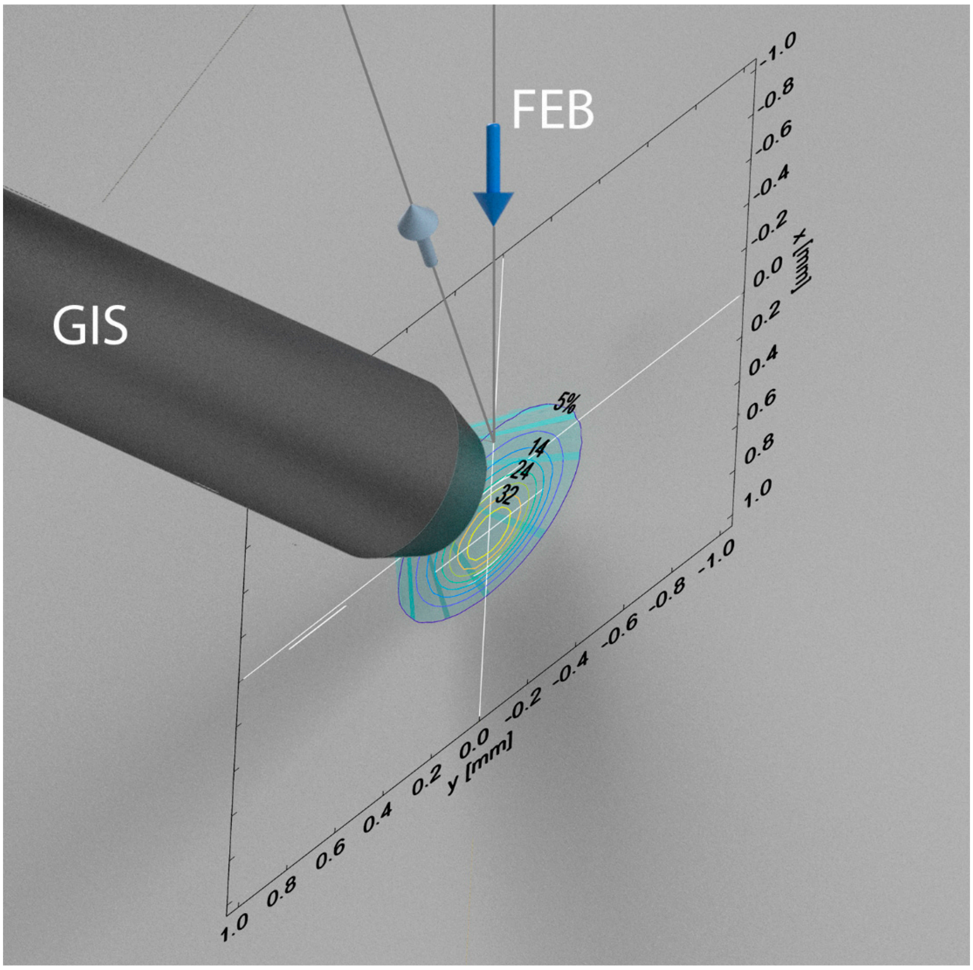

Figure S5. Simulated impinging gas flux in the FEBiMS arrangement. The gas injection nozzle is placed 200 µm above the substrate. The FEB impinges vertically (blue arrow) and secondary electrons are extracted to the SE detector (gray arrow).
